# Effective BAC clone anchoring with genotyping-by-sequencing and Diversity Arrays Technology in a large genome cereal rye

**DOI:** 10.1038/s41598-018-26541-y

**Published:** 2018-05-30

**Authors:** Ewa Borzęcka, Anna Hawliczek-Strulak, Leszek Bolibok, Piotr Gawroński, Katarzyna Tofil, Paweł Milczarski, Stefan Stojałowski, Beata Myśków, Małgorzata Targońska-Karasek, Agnieszka Grądzielewska, Miłosz Smolik, Andrzej Kilian, Hanna Bolibok-Brągoszewska

**Affiliations:** 1 Department of Plant Genetics, Breeding and Biotechnology, Warsaw University of Life Sciences - SGGW, Nowoursynowska 159, 02-776 Warsaw, Poland; 2 Department of Silviculture, Warsaw University of Life Sciences - SGGW, Nowoursynowska 159, 02-776 Warsaw, Poland; 3 Department of Plant Genetics, Breeding and Biotechnology, West-Pomeranian University of Technology, Slowackiego 17, 71-434 Szczecin, Poland; 4 Institute of Genetics, Breeding and Biotechnology, University of Life Sciences in Lublin, Akademicka 15, 20-950 Lublin, Poland; 5 Diversity Arrays Technology Pty Ltd, University of Canberra, Kirinari st, ACT 2617 Bruce, Australia

## Abstract

Identification of bacterial artificial chromosome (BAC) clones containing specific sequences is a prerequisite for many applications, such as physical map anchoring or gene cloning. Existing BAC library screening strategies are either low-throughput or require a considerable initial input of resources for platform establishment. We describe a high-throughput, reliable, and cost-effective BAC library screening approach deploying genotyping platforms which are independent from the availability of sequence information: a genotyping-by-sequencing (GBS) method DArTSeq and the microarray-based Diversity Arrays Technology (DArT). The performance of these methods was tested in a very large and complex rye genome. The DArTseq approach delivered superior results: a several fold higher efficiency of addressing genetic markers to BAC clones and anchoring of BAC clones to genetic map and also a higher reliability. Considering the sequence independence of the platform, the DArTseq-based library screening can be proposed as an attractive method to speed up genomics research in resource poor species.

## Introduction

Bacterial artificial chromosome (BAC) libraries are an indispensable tool in genomics and genetics research. BAC clones arranged into a minimum tilling path are a foundation for the clone-by-clone genome sequencing strategy^1–3^, while paired BAC-end sequences are useful for validating the assembly in case of whole genome shotgun sequencing^4–6^. Identification of relevant genes often relies on the use of BAC libraries during positional cloning^7–9^ or in other approaches^10,11^.

BAC library screening and the resulting identification of clones harboring specific markers/nucleotide sequences is an essential task and a prerequisite for many applications. Initial screening techniques, which relied on hybridization of pooled overgo probes/cDNA clones to BAC clones immobilized on high-density filters^12,13^ or involved PCR assays on multidimensional BAC pools^14,15^, were time-consuming and low-throughput. Over the years several strategies were developed to overcome these limitations and to enable highly-parallel BAC library screening and anchoring of BAC clones to genetic maps. Luo *et al*.^16^ proposed a method which deployed a custom 1536 single nucleotide polymorphism (SNP) Illumina GoldenGate genotyping assay and manual PCR. This approach was further optimized by You *et al*.^17^ and Cao and Schmidt^18^ who, respectively, were able to eliminate manual PCR from the experimental design and adopted the method for the application in polyploid genomes. In 2011 Liu *et al*.^19^ published results of a proof of concept experiments on application of an Agilent expression array to BAC library characterization. Both platforms mentioned above have found a large scale application in anchoring of BAC clones/contigs during generation of sequence enriched physical map of barley^20^.

A considerable input of resources needed to gain sufficient sequence information on the respective genome and to identify a suitable set of polymorphism/genes for the development of a high quality array^16,18^ is an obstacle which prohibits a wider application of commercial array platforms^21^. Diversity Arrays Technology (DArT) is a microarray platform for high-throughput genotyping, independent from the availability of sequence information^22^. Since its invention DArT has been utilized for various purposes, such as linkage mapping and germplasm characterization, in many species^23,24^. So far only very preliminary information on the potential of DArT in BAC library screening is available, based on a single study describing application of DArT alongside SSR and EST markers in anchoring of the physical map of wheat chromosome 3B^25^. The efficacy of DArT in analyzes of whole genome BAC libraries has not been tested yet.

Recent years have witnessed a rapid development of massively parallel next-generation sequencing (NGS)-based genotyping technologies, termed genotyping-by-sequencing (GBS)^21,26^. A particularly exciting feature of these methods is the capability for parallel discovery and genotyping of tens of thousands DNA polymorphisms, while a prior sequence knowledge is not a requirement. DArTseq is a GBS method in which genome-complexity reduction is obtained using a combination of two restriction enzymes, tailored for the genome of the species in question. At least one of the enzymes is methylation sensitive, directing the analysis to the hypomethylated, gene-rich genome regions. Genomic representations, called “targets”, obtained after ligation of adapters to the restriction fragments and PCR amplification, are sequenced with the Illumina short read technology. A proprietary analytical pipeline is used to process the sequence reads and identify polymorphisms. DArTseq has been proven to perform very well in genome-wide analyses of various species, delivering up to tens of thousands SNP and presence/absence variation markers (Silico-DArTs)^27–34^. To date neither DArTseq, nor any other GBS platform, has been used for BAC library screening.

Rye (*Secale cereale* L.) is an important cereal in Central and Eastern Europe, characterized by several interesting traits, such as high nutrient use efficiency and very good stress tolerance, of potential importance also for subsequent improvement of wheat (*Triticum aestivum L*.) and barley (*Hordeum vulgare L*.)^35^. Rye genome size is ca. 7.9 Gbp^36^, and repetitive sequences constitute over 90% of the genome^37^.

The aim of the study was to evaluate the performance of DArT and DArTseq platforms in BAC library screening and BAC clone anchoring with the use of a low-coverage, whole-genome BAC library of the large and complex rye genome and a 3-D clone pooling strategy.

## Results

### DArT/DArTseq marker - BAC clone addressing

In total we addressed DArT or DArTseq markers to 21,639 BAC clones with cumulative length of ca. 2.6 Gbp. Nine settings (detailed in Supplementary Table S1) were tested for the identification of reliable DArT marker – BAC clone assignments. Depending on the setting from 7,464 to 2,066 DArT-BAC addresses were obtained. At the least stringent setting (T1), 4,482 DArT markers were assigned to 2,434 BAC clones. At the most stringent settings (T9), 2,066 DArTs were addressed to 972 BACs. All obtained DArT-BAC addresses are listed in Supplementary Dataset S1. In result of GBS analyses, 221,122 potential DArTseq-BAC addresses were identified. From these addresses we selected 53,370 addresses involving 41,438 rye DArTseq reference sequences (rye DArTseq markers). These markers, ranging in length from 20 to 69 bp (average 56, median 64), were addressed to 19,695 BAC clones (addressing setting TS1). The main reason for dropping from the further analysis the remaining sequences, which did not have “permanent” DArTseq names, was (i) the fact that those markers are likely to represent higher copy number sequences, and also (ii) the inability to link them to the previously reported (and genetically mapped) markers. The numbers of addresses involving markers which occurred only once or twice (setting TS2), or only once (setting TS3) in the screened fraction of the library were, respectively, 46,499 and 32,217. A description of the settings used and the summary of DArTseq-based library screening is given in Supplementary Table S2. All DArTseq-BAC addresses obtained are listed in Supplementary Dataset S2, while the sequences of DArTseq markers involved are given in Supplementary Dataset S3. A statistical analysis (Wilcoxon rank sum test) showed a highly significant difference between DArTseq and DArT-based screening results (Supplementary Table S3).

**Figure 1:**
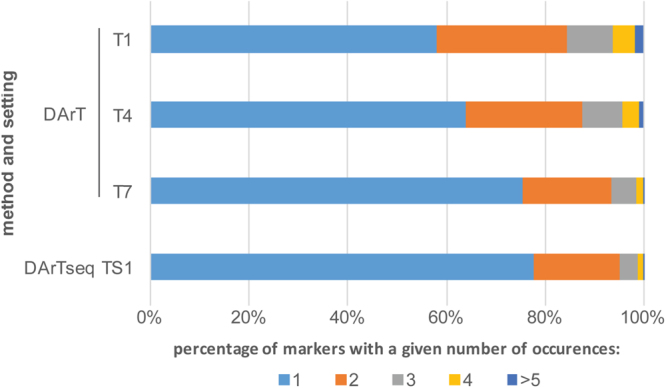
Percentages of DArT and DArTSeq markers with a given number of occurrences in the library screening data at different settings.

The majority of DArT and DArTseq markers occurred only once or twice in the identified addresses, with the proportion of DArT markers which occurred only once raising with the stringency of the marker selection settings up to ca. 75%. A similar value (ca. 78%) was observed for DArTseq markers Fig. 1). While the numbers of identified DArT-BAC and DArTseq-BAC addresses diminished with the raising stringency of the settings, the average number of markers per BAC clone was fairly consistent and ranged from 1.8 to 2.2. A single genetic marker was assigned to ca. 40–50%, two markers to ca. 20%, three markers to ca. 10% of the BAC clones. A slight increase in the proportion of BACs with a single marker was observed at more stringent settings for both DArT and DArTseq markers (Supplementary Fig. S1).

## BAC Clone Anchoring

### DArT marker-mediated anchoring

**Figure 2:**
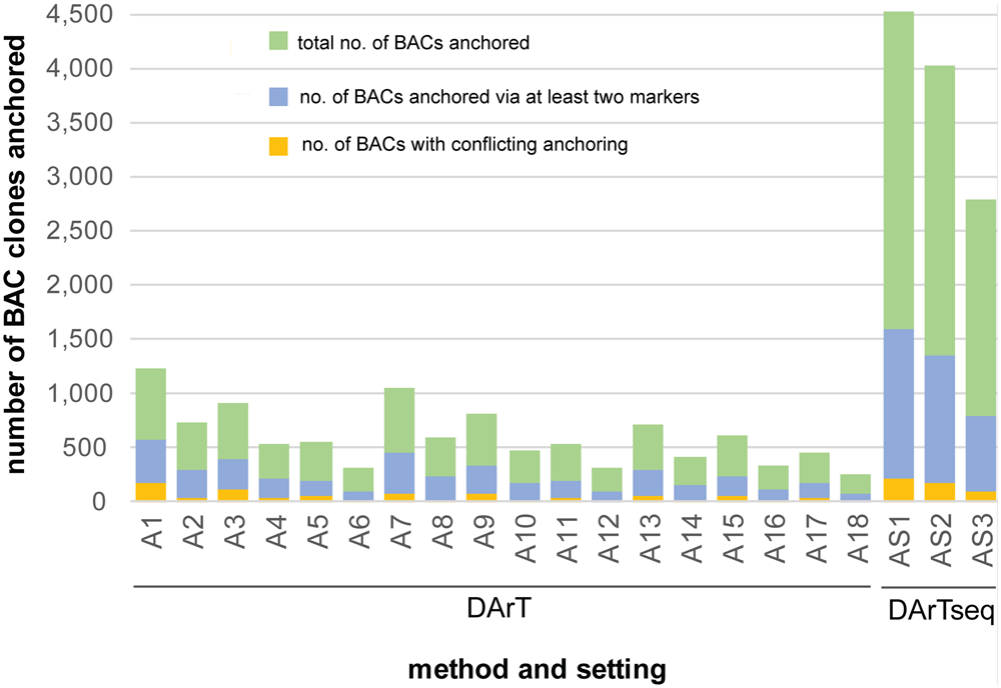
Numbers of BAC clones anchored per method and setting. Top of the blue bar indicates the number of BACs with at least two anchor markers, top of the orange bar indicates the number of BACs with conflicting anchoring.

The integrated map produced using LPMerge (Supplementary Dataset S4) comprised 4,230 DArT markers at 1,296 map positions (average distance 0.88 cM). Since inconsistencies between DArT-based maps of different rye crosses were observed^38,39^, we identified a subset of 2,354 “doubly mapped” DArT markers (markers that were genetically mapped to the same chromosome in at least two crosses) for a more reliable BAC anchoring. The summary of anchoring results, including a description of the anchor markers selection settings, is given in Supplementary Table S4. In total 1,688 mapped DArT markers occurred in the DArT-BAC addresses established at the least stringent setting, 892 of them were “doubly mapped” markers. In result 1,240 BAC clones (Fig. 2) were anchored to 704 map positions, with the average distance between anchoring positions equal to 1.87 cM (anchoring setting A1). A subset of 733 BAC clones was anchored with “doubly mapped” markers to 378 map positions, with the average distance of one anchoring point per 2.98 cM (setting A2). At the most stringent settings, the numbers of anchored BACs were reduced to 459 (via all mapped markers, setting A17) and 258 (via “doubly” mapped markers). The percentage of BAC clones anchored via at least two DArT markers was fairly constant across the settings (38.4% on average). Lists of BAC clones anchored at different settings are given in Supplementary Dataset S4.

### DArTseq marker-mediated anchoring

In total 32,699 DArTseq markers were placed in the composite map at 11,393 positions (Supplementary Dataset S5). Due to a different map compilation procedure used for DArTseq data (one map used as a reference, with markers from the remaining maps placed relative to the framework of the reference map), we did not identify a set of “doubly mapped” markers for DArTseq-mediated anchoring. Map positions were available for 5,436 DArTseq markers involved in the DArTseq-BAC addresses. In result 4,525 BAC clones (Fig. 2) were anchored to 3,324 genetic map positions, with the average distance between anchoring points equal to 0.47 (setting AS1). At the most stringent setting (AS3) 2,783 BAC clones were anchored to 2,544 map positions, at an average distance of 0.62 cM. The summary of DArTseq-based anchoring, including a description of the settings used, and lists of BAC clones anchored are given, respectively, in Supplementary Table S2 and Supplementary Dataset S5.

Using both methods we anchored in total 5,631 BAC clones (cumulative length of ca. 681 Mb, corresponding to ca. 5.4 *Arabidopsis* genomes).

## Reliability of Addressing

### Sequence-based verification

We obtained sequences of seven BAC clones from the super pools (SPs) which were genotyped with both DArTseq and DArT markers and of one BAC clone from a SP genotyped with DArT markers only. The obtained sequences ranged in length from 94,983 to 145,571 bp (122,016 bp on average) and were deposited in GenBank under accession numbers, respectively, MG646011-MG646017 and MG669358. Each of them is a preliminary draft sequence consisting of several scaffolds. In total 64 DArT and 91 DArTseq markers were addressed to these BAC clones at the least stringent settings (T1 and TS1, respectively). Good quality sequences were available for 56 of the DArT markers in question. Most of these sequences (51) were obtained during an earlier study^38^. Five additional DArT clones were sequenced during the present study and the sequences were submitted to GenBank (accession numbers KS718581- KS718581). On average 86.4% (and up to 93.3% at setting T6) of the expected DArT markers were confirmed in the sequenced BAC clones. The average alignment length was 94.5% of the DArT marker sequence length, while the average sequence identity was 92.4%. In the case of DArTseq markers, from 98.8% up to 100% of markers sequences, depending on the setting, resulted in significant alignments to the respective BAC clone sequences, with the average alignment length of over 99.95% of the DArTseq and average sequence identity also over 99.95%. Detailed results of BLAST similarity searches are given in Supplementary Dataset S6. Statistical analysis (Wilcoxon signed rank test) of average identity and average percentage alignment length values calculated for individual BAC clones showed significant differences between DArT- and DArTseq-derived results (Supplementary Dataset S6). Additionally, we calculated variance in average identity and average percentage alignment length for all possible combinations of two, three, four, etc., of the sequenced BAC clones. We observed over 10-fold reduction of variance when increasing the number of BAC clones from two to seven (or eight in case of DArT) (Supplementary Fig. S2). As the analyzed parameters approach “0” at BAC clone numbers below the numbers used in this paper, we conclude that we selected a fairly representative sample size (seven/eight BAC clones), especially given financial constraints on further sample size increases.

We also observed that DArT markers sharing sequence similarity were consistently assigned to the same BAC clones. Among all 1,203 rye DArT marker sequence contigs described by Gawronski *et al*.^38^, we identified 385 contigs which were fully represented - i.e. all 1,148 member DArT markers from these contigs occurred in the library screening results at the least stringent setting. These DArT markers were assigned to 710 BAC clones in total. All DArT markers from a given contig were assigned to the same BAC clone in 59% of cases, and over 50% DArT markers from a given contig were assigned to the same BAC clone in 70% of cases analyzed. For example, the DArT marker sequence contig 410782_con consists of ten DArT marker sequences. Nine markers from this contig were addressed to each of three BAC clones: S21p2J17, S23p7E6, S24p5A24, with a different DArT marker from the contig missing in each of these BAC clones. This data indicates that stringent criteria were chosen for defining DArT-BAC assignments, which resulted in a moderate level of false negatives. It may also reflect slight differences in the quality of arrays used in different experiments - in total four DArT genotyping experiments were performed with samples from four SPs in each experiment. Finally, small inconsistencies observed among DArT markers from the same sequence contig could be attributed to the residual, but noticeable differences in the length and sequence of representatives of the same contigs.

### Genetic map positions-based verification

Inspection of anchoring results revealed anchoring conflicts (markers from different chromosomes assigned to the same BAC clone). For DArT marker-based anchoring the percentage of BAC clones with anchoring conflicts was 31.57% at setting A1. This value decreased with the rising stringency of anchor marker selection down to 8–11% (Fig. 2). The most pronounced reduction of anchoring conflicts (by ca. 50%) was observed when only “doubly mapped” markers were used in anchoring. In most cases (over 85%, up to 97.6%) the anchoring markers assigned to a BAC clone were spread over a genetic map segment shorter than 5 cM. BAC clones containing markers from the same chromosome, but spread over a distance of more than 20 cM constituted from 10.6% down to 0.44% of the BAC clones analyzed. Again, the biggest reduction in number of such BAC clones (by at least ca. 40%) was obtained when anchoring was based only on “doubly mapped” markers (Supplementary Table S3, Supplementary Fig. S3). In the case of DArTseq-based anchoring, the percentage of BAC clones with anchoring conflicts was relatively consistent across stringency levels and ranged from 13.3% down to 11.4%. For approximately 47% of BAC clones containing at least two mapped DArTseq markers from the same chromosome the respective markers were spread over a distance less than 5 cM, while DArTseq markers located on the genetic map more than 20 cM apart were addressed to ca. 22% of BAC clones, irrespective of the settings (Supplementary Table S4, Supplementary Fig. S3).

## Discussion

In the present study we tested the efficiency and reliability of a BAC library screening approach involving the use of a microarray-based technology DArT and a GBS technology DArTseq. To our knowledge it is the first report on application of both methods to an analysis of a whole genome BAC library.

A key feature of both DArT and DArTseq is a genome complexity reduction method relying on the use of a methylation sensitive endonuclease for targeting single copy genome regions. This feature could be a potential drawback during BAC library screening. Since DNA methylation is not maintained in BAC clones^40^, the DArT/DArTseq genotyping procedure could in theory identify BAC clones bearing additional copies of a genetic marker (if present in the genome), which are not recognized during genotyping of plant genomic DNA because of methylation at the relevant restriction enzyme cutting sites. Such situations would in turn result in problems during anchoring of the BAC clones to genetic maps, due to a difficulty in identifying the BAC clone with the “true” genetic marker – the marker which was recognized and mapped based on plant genomic DNA genotyping. However, the existing data shows explicitly that this difference in the methylation status could have only a negligible influence on the quality of DArT/DArTseq-BAC clone addressing, owing to the fact that markers generated using both DArT microarray technology and DArTseq GBS technology are predominately single copy markers. Analyses deploying whole genome reference sequences demonstrated that 97.1% of DArT clone sequences aligned to a single locus in the *Eucalyptus grandis* genome sequence^41^, while, respectively, 82% and 94% of DArTseq marker sequences mapped to a single site in the oil palm^42^ and tomato genomes^43^. Unfortunately, a rye reference genome sequence is not available yet, therefore, we were not able to obtain such data for rye DArT and DArTseq markers. To further reduce the possible influence of the difference in the methylation status, the experimental design implemented in the present study (discussed in the following section) was focused on identification of single copy markers for the establishment of genetic marker-BAC clone assignments.

Several methodical measures were taken to ensure a high reliability of DArT- and DArTseq-BAC addressing. Firstly, a 3-D polling scheme based on seven plate SPs and involving a creation of three types of matrix pools was implemented for library screening. While, over the years, several pooling schemes were developed^14,17,25^, we have chosen this particular scheme due to several advantages it offers: (i) pooling is straightforward to perform, reducing thus the risk of errors, (ii) BAC clones from a SP are represented only by 23 samples, which increases the screening throughput, (iii) every BAC clone from a SP is present in two matrix pools of each type, providing an additional internal control for the quality of both sample preparation and genotyping, (iv) a relatively small fraction of the genome is contained in a SP (ca. 1/25 of the rye genome in this study), which lowers the probability that a given single copy genome region will be present in more than one BAC from a given SP, and results in a higher number of unequivocal genetic marker – BAC clone assignment^16,17^. Furthermore, each SP was processed separately during inoculation of bacterial cultures, pooling, and DNA isolation - to reduce the risk of cross-contamination, and also during data processing - to effectively select for single copy markers. Secondly, to maximize the number of the recovered unambiguous genetic marker-BAC clone assignments, we strived to ensure an equal representation of the BAC clones in the BAC pools and DNA samples: (i) BAC clones were grown separately in 384-well plates and not cultured together after pooling to avoid a bias resulting from different growth rates of individual clones, (ii) BAC DNA isolation was done using a manual method, which resulted in high BAC DNA yield and eliminated thus the need of DNA amplification prior to genotyping and a possible bias in sequence representation due to incomplete/inaccurate amplification or contamination^44,45^. In addition, marker selection criteria based on values of marker quality parameters were used in the case of DArT markers, while in the case of DArTseq assays only the sequence tags previously discovered in analyses of plant genomic DNA and defined as rye DArTseq markers were retained for addressing - to eliminate sequences from repetitive regions and contaminations. For both methods we also separately reported data involving only those markers which occurred only once or twice, or only once, in the library screening results, aiming at stringent selection of single copy markers for even more reliable genetic marker-BAC clone assignments.

The implemented experimental design resulted in a preferential identification of single copy markers – depending on the settings between ca. 84 and 95% of the markers involved in the DArT- and DArTseq-BAC addresses occurred once or twice in the whole dataset, which is consistent with the genome coverage of the library fraction analyzed. These data are also in good agreement with the results of PCR based screening of the same library – Bakera *et al*.^10^ screened the whole SccPanALLhA library for five single copy genes and identified three positive BAC clones for each of four genes and two positive BAC clones for the fifth gene.

Using several approaches we demonstrated that the reliability of the obtained DArT- and DArTseq-BAC clone assignments was very high: (i) we confirmed the presence of 86.4% of the expected DArT markers and of 98.8% of the expected DArTseq markers in the sequences of the respective BAC clones, (ii) DArT markers sharing sequence similarity were addressed consistently to the same BAC clones, (iii) multiple genetically mapped markers addressed to the same BAC clone had adjacent positions on genetic maps. In the case of the DArTseq-based screening, the average distance spanned on a genetic map by markers from a single BAC clone was larger than in the case of DArT markers, but this issue is most probably related to genetic map quality and will be discussed further later on. The fact that not all markers could be confirmed in the BAC clone sequences can be explained by the preliminary status of the BAC clone sequences used. An incomplete confirmation of the expected markers was also reported by Liu *et al*.^19^. The authors were able to confirm 84.5% of the addressed unigenes in the Illumina 2GS sequencing reads obtained from the total DNA of the respective SP and indicated a variable sequence coverage as the most probable reason for the lack of confirmation for the remaining unigenes. The lower confirmation rate obtained in our study for DArT markers (compared to DArTseq) is probably related to technical errors which occurred during preparation of DArT clones for sequencing and during sequence processing.

The high percentage of markers confirmed in the sequenced BAC clones, which demonstrates a very high reliability of the obtained DArT- and DArTseq-BAC addresses, is in contrast with a relatively high percentage of BAC clones with anchoring conflicts. BAC clones containing markers from distant parts of the same chromosome were also identified. These results can be explained by both methodological errors and biological phenomena such as: (i) BAC library related issues – existence of chimeric clones and presence of multiple clones in some wells of the library plates, (ii) genetic map related issues – erroneous assignments of markers to chromosomes and inconsistent marker ordering due to, for example, small mapping population sizes or differences in recombination frequencies between markers in different crosses^42,46,47^, and (iii) structural variation among parental lines of the mapping populations. Cross contamination during library handling, resulting in the presence of a BAC clone in several adjacent wells, can be excluded as a potential factor which compromised anchoring since at the initial stage of data processing we introduced a selection against genetic marker-BAC clone assignments occurring multiple times in a given SP. We tested several settings with the aim of reducing the number of anchoring conflicts, and, as expected, the most pronounced reduction was observed when only “doubly mapped” markers were used as anchors. This outcome is consistent with the reported discrepancies in the rye genetic maps and small sizes of most populations used in mapping^38,39,48^. Finally, we were able to reduce the number of anchoring conflicts to ca. 8–10% in DArT based anchoring. Coinciding values were obtained for DArTseq-based anchoring, providing a further indication of a very similar performance of both platforms. A comparable level of anchoring conflict (8%) was reported by Cvicova *et al*.^49^ who tested a physical map anchoring approach deploying (i) NGS-sequencing of BAC clone pools from a wheat 3DS BAC library and (ii) a resource based on the synteny of grass genomes (GenomeZipper) instead of a genetic map. We hypothesize that these persisting anchoring conflicts reflect mostly BAC library-related issues or genome structural variation. Although there is no direct evidence on structural variation in rye yet, emerging data from other plants demonstrates that the extent of this type of variation can be considerable^2,50,51^.

In comparison to the preliminary results of DArT-based screening of a 3B BAC library of wheat^25^, we achieved a much higher addressing efficiency, with a more than ten times bigger number of DArT markers successfully addressed per given number of BAC clones analyzed. This higher success rate is most probably a result of an improved methodology. The key modifications included: a different pooling scheme, pooling procedure without common growth of pooled BAC clones, and elimination of BAC pool DNA amplification step prior to genotyping. With respect to the number of BAC clones addressed per SPs, the efficiency was higher in the study deploying an Agillent microarray^19^ than in the DArT-based screening described here: 727 versus 152 BAC clones, respectively. On the other hand, the addressing efficiency in terms of the percentage of the probes from the array which were successfully addressed to BAC clones was comparable for both methods – 2.4% for DArT and 3.7% for the Agillent microarray. A point worth stressing is that, compared to other microarray platforms, DArT microarray technology offers clear advantages in terms of cost required to establish the technology and also in cost per genotyping experiment. Irrespective of the efficiency of the method, in the context of research on rye, the biggest value of DArT-based BAC library screening results lays in the fact that a considerable number of studies were conducted to date using the rye DArT panel (see^38^ for the reference list), and through the DArT-BAC assignments obtained we provide means of direct access to many genomic regions of interests identified in those studies.

Consistently with the potential of GBS methods to deliver tens of thousands of markers in a single experiment, DArTseq-based library screening resulted in ca. seven times more genetic marker–BAC clone assignments in comparison to DArT-based screening. The number of BAC clones which were anchored to genetic map via DArTseq markers is admittedly only ca. 3.6 times bigger than the number of BAC clones anchored via DArT markers, however, the saturation of the DArTSeq genetic map was likely the limiting factor. While several DArT maps, including a consensus map, based on mapping populations representing diverse genetic backgrounds, were published to date, the DArTseq-based consensus map of rye is in an early stage of development. The map used in this study was created using data from five interrelated and rather small populations, which negatively influenced the number of segregating markers and the accuracy of mapping. Additionally, the parental lines were genetically distant from the BAC library donor line. It can be assumed that, over time, with the accumulation of data from DArTseq genotyping of diverse rye accessions and populations more DArTseq sequence tags from over 200,000 initially obtained DArTseq-BAC clone assignments will be recognized as rye DArTseq markers and genetically mapped, resulting in a higher efficiency of genetic marker to BAC clone addressing and BAC clone anchoring.

We conclude that the proposed BAC library screening approach, implementing the microarray DArT method or the GBS method DArTseq, results in high-throughput, reliable, straightforward, and cost-effective addressing of genetic markers to BAC clones. The performance of these methods was tested in a very large and complex rye genome. Hence, it can be expected that the proposed approach would produce satisfactory results in most plant genomes. Both methods efficiently anchor BAC clones to genetic maps, with the high quality of genetic maps being crucial for unambiguous anchoring. Fulfilling the potential of GBS methodology, the DArTseq approach delivered superior results: a several fold higher addressing and anchoring efficiency and also a higher reliability. Considering the sequence independence of the platform, the DArTseq-based library screening can be proposed as an attractive method to speed up genomics research in resource poor species.

## Materials and Methods

### BAC Library

The BAC library SccPanALLhA used in this study was constructed from DNA of rye inbred line L318 (S_26_). It consisted of 105,216 clones with the average insert length 121 kb, providing ca. 1.5-fold genome coverage. Details concerning the library construction were described by Bakera *et al*.^10^.

### BAC clone pooling

To increase the screening throughput and to reduce the number of equivocal BAC clone-genetic marker assignments, a 3-D pooling strategy, described in detail by Liu *et al*.^19^, was applied. In this method the library is divided into SPs consisting of seven 384-well plates. During the first stage of pooling, seven plate pools, 16 row pools, and 24 column pools are created for each SP by combining equal amounts of bacterial culture from the respective wells of the seven 384-well plates. In the second stage of pooling, the plate, row, and column pools from the first stage of pooling are combined into five matrix plate pools, eight matrix row pools, and ten matrix column pools, respectively, whereas each row, column or plate pool is contained in two matrix pools of the respective type. The library screening is performed on matrix pools and for each marker present in a single clone out of 2688 (7 × 384) clones in a given SP six positive results should be obtained: in two matrix plate pools, in two matrix column pools, and in two matrix row pools. After deconvolution of the screening results, the coordinates of the positive BAC clone (plate, row, and column) are obtained. BAC clones were grown individually in 384- well plates for 22 hours at 37 °C in 80 µL of LB medium supplemented with 12,5 mg/L chloramphenicol. From each mother plate three daughter copies were inoculated with a 384-pin replicator for the production of plate, row, and column pools, respectively. During the first stage of pooling, 35 µL of bacterial cultures were combined manually, using multichannel pipettes, or with the help of Janus Multiprobe (Perkin Elmer) liquid handling station. Matrix pools were done manually by combining equal volumes of row, column or plate pools.

### BAC DNA isolation

BAC DNA was isolated manually from six mL of pooled bacterial cultures using a modified alkaline lysis method. The key modification involved the substitution of SDS (sodium dodecyl sulfate) with SLS (sodium N-lauroylsarcosine) in the lysis buffer, as proposed by Sinnet *et al*.^52^. After precipitation with 96% ethanol and rinsing with 70% ethanol, BAC pool DNAs were resuspended overnight in 50 µL of 1 × TE buffer at 4 °C and then transferred to −20 °C for storage. Quality and concentration of BAC DNA was assessed using electrophoresis in 0.8% agarose gels and spectrophotometric measurements on NanoDrop2000 (Thermo).

### DArT and DArTseq genotyping of BAC pool DNA

BAC pool DNA samples were shipped for genotyping to Diversity Arrays Technology Pty Ltd (Bruce, Australia). Both DArT and DArTseq genotyping experiments were done in full technical replication. In total 28 SPs (75,264 clones) were genotyped - 12 SPs with DArT microarray technology, 12 SPs with DArTseq platform, and four SPs using both methods. Based on the average insert size and the percentage of empty clones of the BAC library SccPanALLhA, ca. 9.04 Gbp of rye DNA was screened in total (ca. 5.17 Gbp with each screening method). Genotyping of BAC pool DNA on the 11,520-clone rye DArT array was carried out essentially as described in^53^. Cut off values of reproducibility (concordance of the genotype call between the replicates) ≥96% and call rate (percentage of effective scores) ≥80%, routinely used during genomic DNA genotyping, were applied to identify high quality DArT markers for subsequent data processing. In addition, we tested two more stringent sets of marker quality thresholds: (i) reproducibility = 100%, call rate ≥95%, and (ii) reproducibility = 100%, call rate = 100%. For DArTseq analyses the protocol reported by Al-Beyroutiova *et al*.^54^ was followed, with minor modifications: sequencing of representations obtained from BAC clone pool DNAs was carried out on Illumina HiSeq2500 and only the Silico-DArT (presence or absence of restriction fragments in representation) calling algorithm of DArTSoft14 was used for marker identification. For further analyses we selected only those Silico-DArTs which were identified by Diversity Arrays Technology Pty Ltd service as rye markers and are stored in DArTdb database as Rye DArTseq reference sequences.

### Clone deconvolution

Data from each SP was analyzed separately. First, by selecting markers with exactly six positive scores in 23 matrix pools: two in matrix plate pools, two in matrix row pools, and two in column pools, we identified DArT markers occurring only once in a given SP. Then, Microsoft Excel formulas were used to identify the plate and the well (row and column information) with the BAC clone bearing the marker in question.

### Selection against multicopy markers

To increase the reliability of anchoring, we identified a subset of markers occurring only once or twice in total in all SPs analyzed. Since (i) the total genome coverage of the library used was ca. 1.5-fold and (ii) no attempt was made at random selection of library plates into SPs, it is likely that a given rye genome region was represented twice in the SPs analyzed and that genetic markers which occurred once or twice in the screened fraction of the library are very likely to be single copy markers. Addressing based solely on markers occurring only once in the screened fraction of the BAC library was also performed.

### Anchoring of BAC clones to genetic maps

For the DArT marker-mediated anchoring of BAC clones an integrated map was produced with LPmerge^55^, following the procedure described by de Miguel *et al*.^56^. Data from six individual DArT-based linkage maps: five maps reported by Milczarski *et al*.^48^ and the HYB201 x HYB202 map by Hackauf *et al*.^39^ was used. During merging maps were given weight according to population sizes. Anchoring via DArTseq marker was based on a composite map generated with Biomercator V4.2 software^57^ from individual linkage maps of four populations sharing the female parent: 541 × 2020LM (92 RILs)^58^, 541 × DD1 (91 F_2_ individuals), 541 × S44 (89 F_2_ individuals), and 541 × EM1 (88 F_2_ individuals). The only published so far DArTseq-based map of rye – the map of the cross 541 × 2020LM^58^ – was used as a reference map during the map compilation procedure. Construction of the genetic maps of the crosses 541 × DD1, 541 × S44, and 541 × EM1 will be described separately.

### Validation of the BAC clone-DArT and BAC clone-DArTseq marker assignments

To test the reliability of DArT/DArTseq - BAC addressing we used sequence-based and genetic map location-based approaches. Firstly, sequence similarity searches using BLASTN algorithm^59^ were applied to confirm the presence of the expected DArT and DArTseq markers in the sequences of selected BAC clones from the genotyped SPs. For DArT markers e-value < 0.00001, percentage of identical matches (identity) ≥70%, and alignment length ≥200 were used as a threshold. For DArTseq markers the cutoff values were: e-value < 0.00001, identity ≥75%, alignment length ≥75% of DArTseq marker sequence length. Average identity and average percentage alignment length (alignment length × 100%/marker sequence length) were also calculated for each BAC clone, for both DArT and DArTseq markers. Additionally, information from a sequence based characterization of 6,177 rye DArT markers^38^ was used to check if DArT markers sharing sequence homology were addressed to the same BAC clone. Specifically, we took advantage of the fact that there is ca. 40% redundancy in the DArT rye genotyping panel and that 3,643 rye DArT markers were assigned to 1,203 contigs based on sequence similarity^38^. Secondly, in cases where multiple genetically mapped DArT or DArTseq markers were addressed to a BAC clone, the genetic map locations of the respective markers were compared. We analyzed (i) if multiple markers assigned to a BAC clone were located on the same chromosome on the integrated map and (ii) what distance did the markers span on the genetic map.

### BAC clone sequencing

Eight BAC clones included in the genotyped SPs were shipped to a commercial sequencing facility at the Institute for Biochemistry and Biophysics (Warsaw, Poland) for sequencing (Illumina MiSeq platform) and sequence assembly.

### Data availability

Nucleotide sequences of DArT markers and BAC clones used in this study can be retrieved from GenBank. The datasets generated during and/or analyzed during the current study are available online as Supplementary Tables S1–S4, Supplementary Datasets S1–S6 and Supplementary Figures S1–S3, and from the corresponding author on reasonable request.

## Electronic supplementary material

Supplementary Information

Dataset 1

Dataset 2

Dataset 3

Dataset 4

Dataset 5

Dataset 6
