# Supplementary material for: Effective BAC clone anchoring with genotyping-by-sequencing and Diversity Arrays Technology in a large genome cereal rye
Source: Sci Rep. 2018 May 30;8:8428. doi: 10.1038/s41598-018-26541-y (PMC5976670; doi:10.1038/s41598-018-26541-y)
Supplement: Supplementary file 1 — Supplementary Information [file 41598_2018_26541_MOESM1_ESM.pdf]

Effective BAC clone anchoring with genotyping-by-sequencing and Diversity Arrays Technology in a large genome cereal rye

Ewa Borzęcka, Anna Hawliczek-Strulak, Leszek Bolibok, Piotr Gawroński, Katarzyna Tofil, Paweł Milczarski, Stefan Stojałowski, Beata Myśków, Małgorzata Targońska-Karasek, Agnieszka Grądzielewska, Miłosz Smolik, Andrzej Kilian, Hanna Bolibok-Brągoszewska

Supplementary Information

Supplementary Table S1. Summary of DArT-based library screening results obtained at different settings.

**Stringency settings:**	T1	T2	T3	T4	T5	T6	T7	T8	T9
1. Selection based on marker quality:									
a) reproduciblity 96, call rate 80	X	X	X						
b) reproduciblity 100, call rate 95				X	X	X			
c) reproduciblity 100, call rate 100							X	X	X
2. Selection against mutlicopy markers, based on no. of occurrences of a given DArT marker within the screened library fraction									
a) DArTs with any no. of occurrances	X			X			X		
b) DArTs occurring only once or twice		X			X			X	
c) DArTs occurring only once			X			X			X
**DArT-BAC clone addressing results:**									
No. of DArT-BAC addresses	7,464	4,954	2,602	5,864	4,244	2,432	3,638	3,064	2,066
No. of DArTs involved	4,482	3,778	2,602	3,809	3,338	2,432	2,742	2,565	2,066
No. of BAC clones involved	2,434	1,867	1,171	2,163	1,744	1,149	1,486	1,316	972
No. of DArTs / BAC clone	1.8	2.0	2.2	1.8	1.9	2.1	1.8	1.9	2.1

Supplementary Table S2. Summary of DArTseq-based library screening and BAC clone anchoring results obtained at different settings.

**Marker selection criteria**	**rye DArTseq markers with any no. of occurrences**	**rye DArTseq markers occurring only once or twice**	**rye DArTseq markers occurring only once**
**DArTseq-BAC addressing settings:**	**TS1**	**TS2**	**TS3**
No. of DArTSeq-BAC addresses	53,370	46,499	32,217
No. of DArTseqs involved	41,438	39,357	32,217
No. of BAC clones involved	19,694	18,482	14,845
No. of DArTseqs/BAC clone	2.1	2.1	2.2
**DArTseq-mediated BAC anchoring settings:**	**AS1**	**AS2**	**AS3**
No. of anchored DArTseq-BAC addresses	6,847	6,208	4,044
No. of anchoring DArTseqs	5,436	5,127	4,044
No. of anchored BAC clones	4,525	4,022	2,783
No. of BAC clones anchored via at least two DArTseqs	1,602	1,346	799
No. of BAC clones with anchoring conflicts	213	169	91
% of BAC clones with anchoring conflicts	13.3	12.6	11.4
No. of BAC clones with at least two DArTseqs from the same chromosome	1,507	1,249	740
No. of BACs with DArTseqs spanning less than 5 cM	713	598	356
% of BACs with DArTseqs spanning less than 5 cM	47.3	47.9	48.1
No. of BACs with DArTseqs spanning over 20 cM	336	282	159
% of BACs with DArTseqs spanning over 20 cM	22.3	22.6	21.5

Supplementary Table S3. DArT and DArTseq-based library screening results by superpool.
Result of Wilcoxon rank sum test for no. of addresses: n = 16, W = 0, p-value = 3.327e-09; Result of Wilcoxon rank sum test for no. of BAC clones involved: n = 16, W = 0, p-value = 1.528e-06

DArT-based screening results (setting T1)			DArTseq-based screening results (setting TS1)		
**Superpool**	**No. of DArT-BAC addresses**	**No. of BAC clones involved**	**Superpool**	**No. of DArTseq-BAC addresses**	**No. of BAC clones involved**
S2	510	154	S9	4107	1406
S3	562	172	S10	2402	1008
S4	452	149	S11	2758	1141
S14	431	139	S14	3619	918
S16	364	124	S15	2265	1026
S17	459	172	S18	3511	1471
S21	393	138	S23	3527	1336
S22	474	166	S25	1857	621
S23	323	103	S26	2118	1086
S24	545	161	S27	4552	1744
S29	485	162	S28	2951	1132
S30	557	169	S31	3572	1251
S31	381	120	S32	2441	1090
S33	479	154	S34	4531	1521
S34	523	179	S35	3301	1255
S37	526	172	S36	5858	1688
**Total**	7464	2434	**Total**	53370	18288

Supplementary Table S4. Summary of DArT-based BAC clone anchoring results obtained at different settings.

**Stringency settings:**	**A1**	**A2**	**A3**	**A4**	**A5**	**A6**	**A7**	**A8**	**A9**	**A10**	**A11**	**A12**	**A13**	**A14**	**A15**	**A16**	**A17**	**A18**
1. Selection based on marker quality:																		
a) reproducibility >96, call rate >80	X	X	X	X	X	X												
b) reproducibility =100, call rate >95							X	X	X	X	X	X						
c) reproducibility =100, call rate =100													X	X	X	X	X	X
2. Selection against mutlicopy markers, based on no. of occurrences of a given DArT marker within the screened library fraction																		
a) DArTs with any no. of occurrences	X	X					X	X					X	X				
b) DArTs occurring only once or twice			X	X					X	X					X	X		
c) DArTs occurring only once					X	X					X	X					X	X
3. Selection based on mapping information reliability:																		
a) all mapped markers	X		X		X		X		X		X		X		X		X	
b) "doubly mapped" markers		X		X		X		X		X		X		X		X		X
**Anchoring results:**																		
No. of anchoring DArTs	1688	892	1454	769	997	519	1428	761	1274	686	934	497	1045	563	984	522	813	421
No. of anchored DArT-BAC addresses	2760	1479	1911	1019	997	519	2148	1136	1614	875	934	497	1358	757	1155	623	813	421
No. of anchored BAC clones	1240	733	916	537	566	323	1058	602	825	474	536	311	719	420	619	347	459	258
No. of BACs anchored via at least two DArTs	567	295	405	214	201	106	458	232	341	184	197	100	292	154	240	125	172	84
% of BACs anchored via at least two DArTs	45.7	40.2	44.2	39.9	35.5	32.8	43.3	38.5	41.3	38.8	36.8	32.2	40.6	36.7	38.8	36.0	37.5	32.6
No. of BACs with conflicting anchoring	179	45	112	29	51	11	87	25	74	20	41	8	68	16	49	15	33	8
% of BACs with conflicting anchoring	31.6	15.3	27.7	13.6	25.4	10.4	19.0	10.8	21.7	10.9	20.8	8.0	23.3	10.4	20.4	12.0	19.2	9.5
No. of BAC with at least two DArTs from the same chromosome	514	282	360	200	183	99	413	226	312	176	179	95	261	152	221	121	158	82
No. of BACs with DArTs spanning less than 5 cM	479	247	315	188	167	95	351	203	285	168	168	93	226	138	199	114	148	80
% of BACs with DArTs spanning less than 5 cM	93.2	87.6	87.5	94.0	91.3	96.0	85.0	89.8	91.3	95.5	93.9	97.9	86.6	90.8	90.0	94.2	93.7	97.6
No. of BACs with DArTs spanning over 20 cM	51	13	28	8	10	3	31	6	15	5	5	1	19	4	13	4	6	2
% of BACs with DArTs spanning over 20 cM	10.6	5.3	8.9	4.3	6.0	3.2	8.8	3.0	5.3	3.0	3.0	1.1	8.4	2.9	6.5	3.5	4.1	2.5

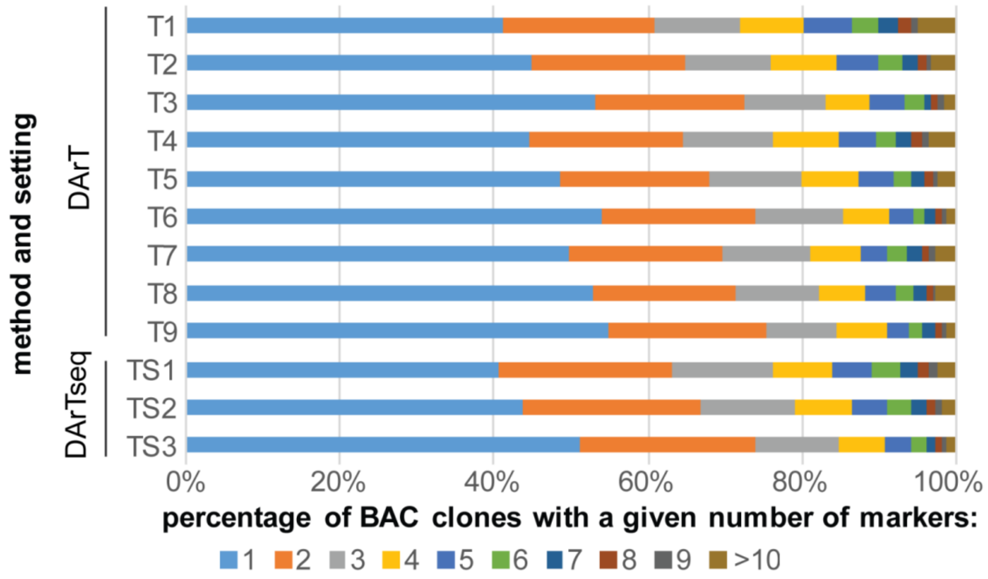

Supplementary Figure S1. Percentage of BAC clones with a given number of marker depending on the method (DArT or DArTseq) and setting

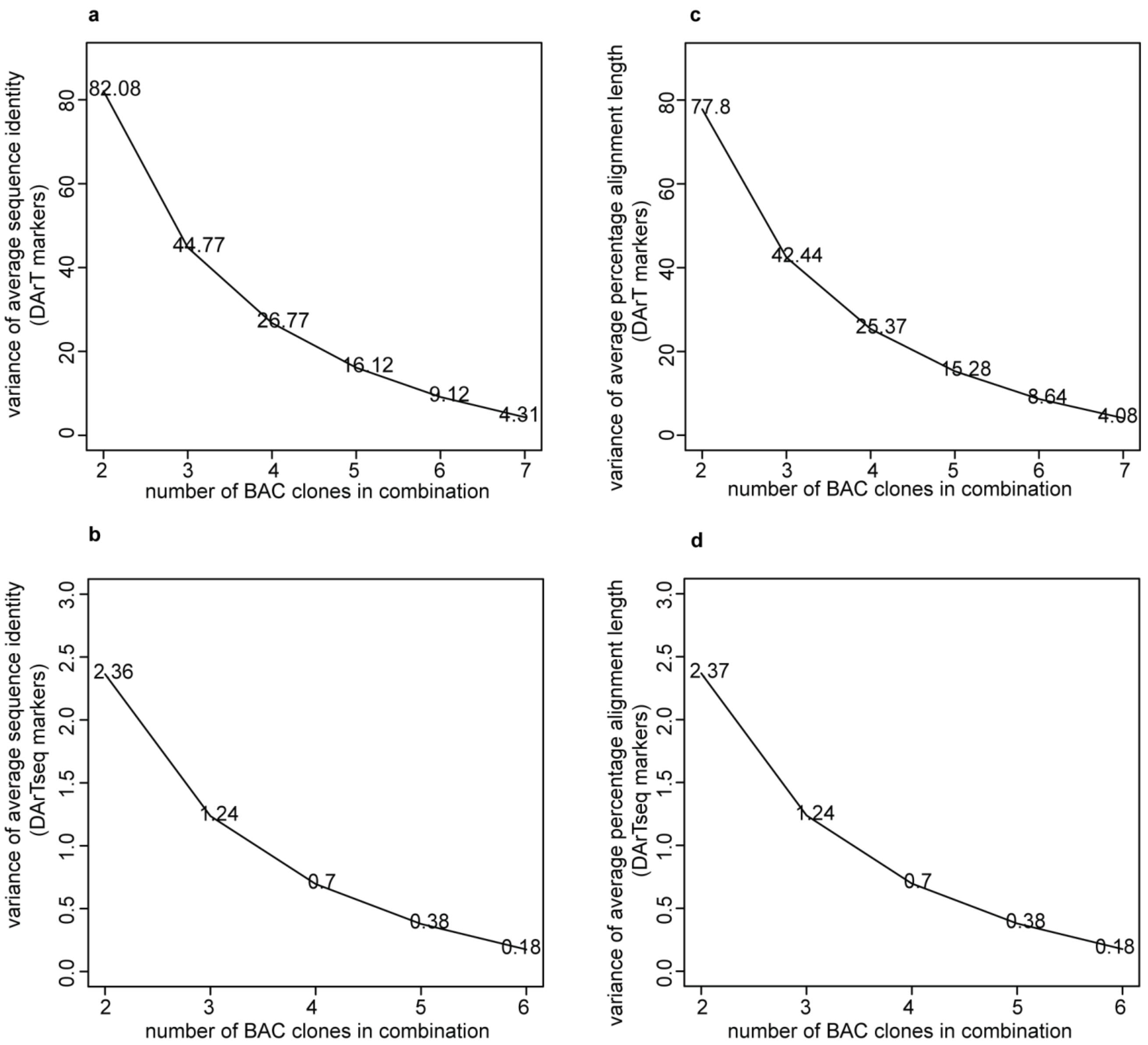

Supplementary Figure S2. Variance in average sequence identity (a, b) and average percentage alignment length (c, d) for all possible combinations of a given number of sequenced BAC clones, based on results of BLAST similarity searches involving DArT (a, c) and DArTseq (b, d) marker sequences.

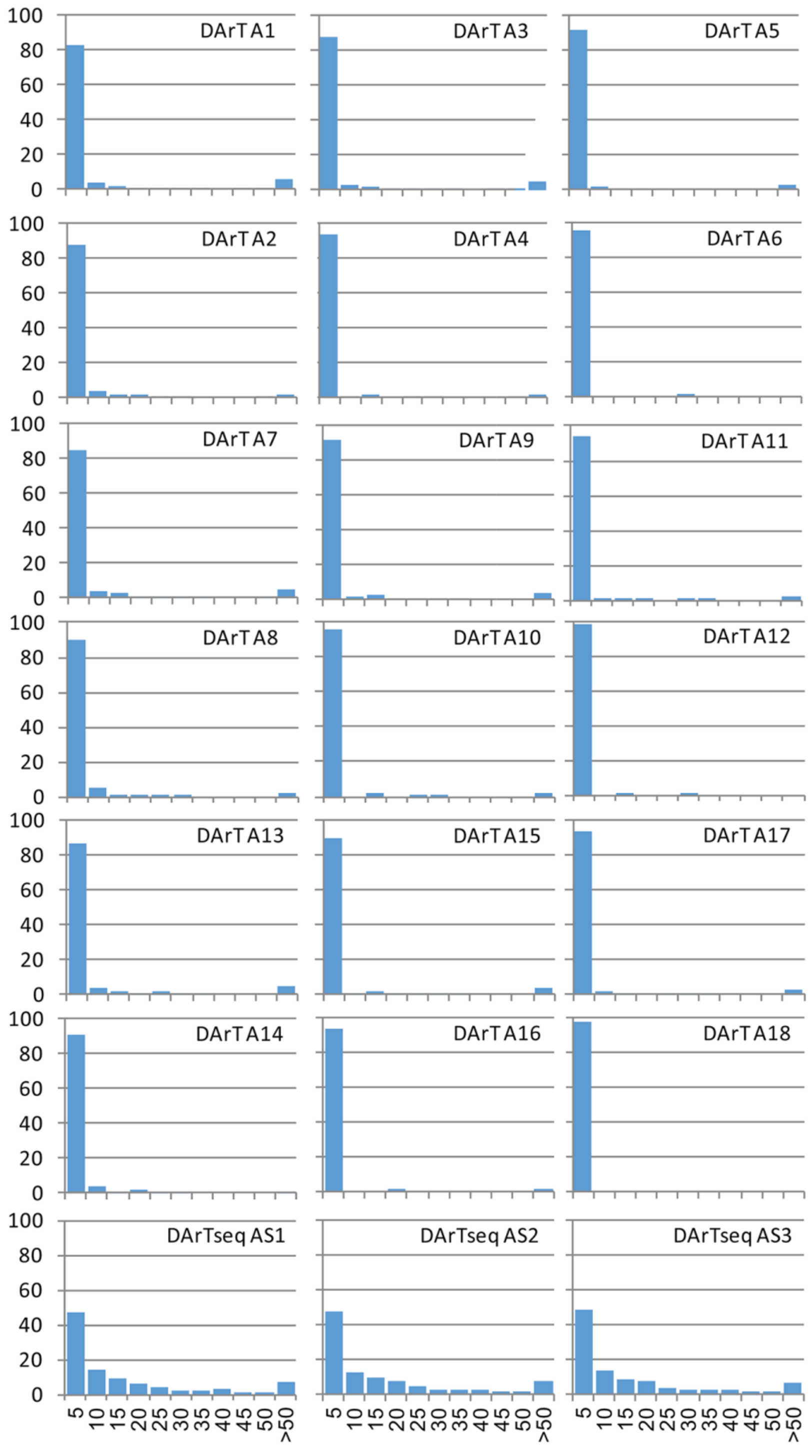

Supplementary Figure S3. Map-based verification of addressing reliability. Percentages of BAC clones containing markers spanning a given distance on genetics maps depending on the method (DArT or DArTseq) and setting (A1-A18 for DArT markers, AS1-AS3 for DArTseq markers).
